# Clinical Effectiveness of Conservative Treatments on Lumbar Spinal Stenosis: A Network Meta-Analysis

**DOI:** 10.3389/fphar.2022.859296

**Published:** 2022-06-06

**Authors:** Xuanwei Chen, Zhizhan Zheng, Jianhua Lin

**Affiliations:** ^1^ The First Affiliated Hospital of Fujian Medical University, Fujian, China; ^2^ Xianyou Country General Hospital, Fujian, China

**Keywords:** visual analog scale, Japanese Orthopaedic Association Score, Oswestry Dysfunction Index, Euroqol Five Dimensions Questionnaire, limaprost

## Abstract

**Objective:** To systematically evaluate the clinical effectiveness of conservative treatments including pharmacological treatments and nonpharmacological treatments on patients with lumbar spinal stenosis.

**Methods:** We searched six electronic databases systematically for randomized clinical trials published between January 2000 and July 2021, including the China National Knowledge Infrastructure, WanFang Data, PubMed, MEDLINE, Embase, and the Cochrane library. The studies focused on the therapeutic effects of pharmacological treatments including calcitonin, antiepileptics, neurotrophic drugs, nonsteroidal anti-inflammatory drugs, Chinese Traditional Medicine, limaprost, and nonpharmacological treatments like physiotherapy for treating lumbar spinal stenosis were included. The outcome was measured using the visual analog scale, Oswestry Dysfunction Index, Japanese Orthopaedic Association Score, and EuroQol Five Dimensions Questionnaire. The quality of eligible studies was assessed by using the Cochrane recommended bias risk assessment tool. Stata was used to conduct the network meta-analysis.

**Results:** A total of 12 randomized control trials with 1,194 patients were included. The network meta-analysis showed that for the visual analog scale, a better therapeutic effect was noted while using Chinese Traditional Medicine and physiotherapy, followed by analgesics drugs and limaprost. Limaprost and calcitonin were better in decreasing the Oswestry Dysfunction Index. In terms of the Japanese Orthopaedic Association Score, the use of traditional Chinese Medicine and limaprost were associated with a better improvement than other treatments. Meanwhile, limaprost combined with analgesics drugs was found to be effective to improve the EuroQol Five Dimensions Questionnaire.

**Conclusion:** Among the commonly used conservative treatments for the treatment of lumbar spinal stenosis, limaprost may have better efficacy in improving the Japanese Orthopaedic Association Score and decreasing the Oswestry Dysfunction Index, with a beneficial effect on decreasing the visual analog scale and improving the EuroQol Five Dimensions Questionnaire.

**Systematic Review Registration:** website, identifier registration number.

## Introduction

Lumbar spinal stenosis (LSS) is a degenerative disease that commonly occurs in the elderly population (Verbiest, 1980), with a high prevalence rate worldwide (LaBan and Imas, 2003). Studies in Japan have shown that the prevalence of symptomatic LSS is 10.0% in the general population (Ishimoto et al., 2012) and approximately 22.5% in the general population of America (Kalichman et al., 2009). In China, the prevalence rate of LSS is approximately 3.9%–11.0% (Letao, 2021), and the rate is expected to increase with the aging of the Chinese population.

Degenerative changes in the intervertebral discs, ligamentum, and synovial joints lead to normal narrowing of the spinal canal and reduction of the internal diameter of the nerve root canal, which irritates or compresses the neurovascular vessels and causes a series of clinical symptoms (Rui, 2020). The typical symptoms of patients with LSS include back pain or intermittent claudication, resulting in the reduction of the patients’ quality of life (QoL) and mobility (Mangone et al., 2020; Rui, 2020).

The major objective of LSS treatment is to reduce pain, improve numbness symptoms, and increase the patient’s mobility. The guideline of LSS (Rui, 2020) recommends surgery as the best treatment for patients with severe LSS, and conservative treatments including pharmacological treatments like medications, nonpharmacological treatments like physiotherapy, lifestyle changes, and others for patients with mild-to-moderate LSS. The commonly used drugs include calcitonin, mecobalamin, gabapentin, celecoxib, loxoprofen, indomethacin, Chinese Traditional Medicine (CTM), and limaprost (Rui, 2020). Although these interventions are commonly used among patients with LSS in the real world, the efficacy of the interventions still remains unknown for related studies are limited. Therefore, our study aims to systematically evaluate the clinical effectiveness of conservative treatments on patients with LSS by network meta-analysis, thus providing evidence for clinical practice.

## Methods

### Searching Strategy

A systematic search was conducted in six electronic databases, including the China National Knowledge Infrastructure (CNKI), WanFang Data, PubMed, MEDLINE, Embase, and Cochrane library from January 2000 to July 2021. The terms used for searching included LSS, the visual analog scale (VAS), the Japanese Orthopaedic Association (JOA) scores, the Oswestry Disability Index (ODI), and 6 min walking test. The detailed searching strategy is shown in the Supplementary Material.

### Inclusion and Exclusion Criteria

The inclusion and exclusion criteria were followed by Patient-Intervention-Control-Outcome methods (da Costa Santos et al., 2007).

The main inclusion criteria were as follows: 1) individuals with LSS were included, excluding those with combined orthopedic diseases such as lumbar disc herniation and fracture, 2) randomized controlled studies (RCTs) focusing on the effectiveness comparison of conservative treatments were eligible to be included in this study, and 3) various outcomes related to the disease were considered to be included, but inclusion was not limited to pain (including leg pain and back pain), disability, physical function, the QoL, adverse events, the satisfaction rate of patients, and recovery. Moreover, the studies focusing on the effects of limaprost in Japanese were included for supplementary, as limaprost was widely used in Japan and generated a lot of clinical evidence.

The studies meeting one of the following criteria would be excluded: 1) publications that are duplicated or studies without full text and 2) studies with a study period of more than 3 months were excluded to maintain consistency.

Conservative treatments in clinical practice were far more complex than the guideline recommendation, so treatment classification was conducted. The pharmacological treatments were categorized as calcitonin, antiepileptics, neurotrophy drugs, analgesics, CTM, and limaprost according to their mechanism and effects. Since nonpharmacological treatments are messy in the real world and the related studies are limited, it is difficult to make further categories of nonpharmacological treatments; nonpharmacological treatments were defined as physiotherapy in this study. Limaprost was the only drug with a specific indication for the treatment of LSS, and its mechanism is to improve the microcirculation of patients with LSS which is different from other drugs (Kurokawa et al., 2011; Kim et al., 2016), making it a separate category. The treatment included in each category were as follows: antiepileptics included gabapentin; neurotrophy drugs included mecobalamin, vitamin B6, etc.; physiotherapy included acupuncture, massage, cupping, etc.; analgesics included nonsteroidal anti-inflammatory drugs (NSAIDs) such as celecoxib and etodolac, pregabalin, etc.; CTM included Biqi granules, Chinese herbal soup, etc. Drug combined with physiotherapy was defined as drug medication. As for the combined treatment of analgesics and neurotrophic drugs, it was classified as analgesics in this study. Moreover, abbreviations were used in tables/figures to be clearer. Lim represents limaprost, Ana represents analgesics drugs, Phy represents physiotherapy, Cal represents calcitonin, Antiep represents antiepileptic drugs, and Neu represents neurotrophic drugs.

As these clinical outcomes are assessed by different scales and methods, a wide range of indicators will be considered for inclusion in this study. Pain was measured using different scales, such as the VAS, Numeric Rating Scale (NRS), and Verbal Rating Scale (VRS). Disability was measured using scales such as the ODI and the Roland Morris Disability Questionnaire (RMDQ). Physical function was measured using the JOA score, max walking distance, pain-free walking distance, and 6-min walking distance. As for QoL, scales like the EuroQol Five Dimensions Questionnaire (EQ-5D) and the short-form health survey questionnaire were commonly used. Recovery was measured by the effectiveness rate and improvement rate. Adverse events were defined as any adverse events reported in the literature, with no restriction on the level of adverse events.

### Study Screening

Following the search, all references of identified studies were imported into the reference management software, NoteExpress. After removing duplicates, two reviewers evaluated the titles and abstracts of the identified studies according to the inclusion and exclusion criteria. Reasons for the exclusion were recorded and reported. For studies included by the title and abstract screening, the full text was reviewed carefully to determine the final inclusion decision. Any disagreement between the two reviewers regarding inclusion and exclusion at each stage of the selection process was resolved through discussions, or by a third reviewer.

### Data Extraction and Quality Evaluation

Two reviewers independently collected data from the selected articles. The corresponding authors were contacted through email to collect detailed information, in case of any uncertainty. The extracted information from the included studies was as follows: 1) the basic information of the study, including the title, author, and year; 2) baseline characteristics of the study participants and the treatment; 3) outcomes and the results; and 4) the risk assessment of the study.

The quality of included studies was assessed by the bias risk assessment tool, which is recommended by the Cochrane Handbook version 6.3 for Systematic Reviews of Interventions (Higgins et al., 2022). This tool includes seven items, including random methods, allocation concealment, blinding researchers and participants, blinding research results, completeness of data, selective reporting of research results, and other biases. These items were classified into low risk, high risk, and unclear based on the specific research content.

### Statistical Methods

A network meta-analysis was conducted by using Stata. For each clinical indicator, the network plot was drawn, respectively, to demonstrate the comparison of interventions. In the network plot, the lines represented the number of included studies and the node represented the number of patients included in the intervention. The thicker the node, the more studies included; the larger the node, the more patients included. As for the effectiveness, it was measured by the ranking of the area under the curve. The larger the area under the curve, the better the effect of the intervention. The node-splitting method was performed to test consistency, and *p* < 0.05 was used as the level of statistical significance. The sensitivity analysis was conducted by using the inverted funnel chart.

## Results

**FIGURE 1:**
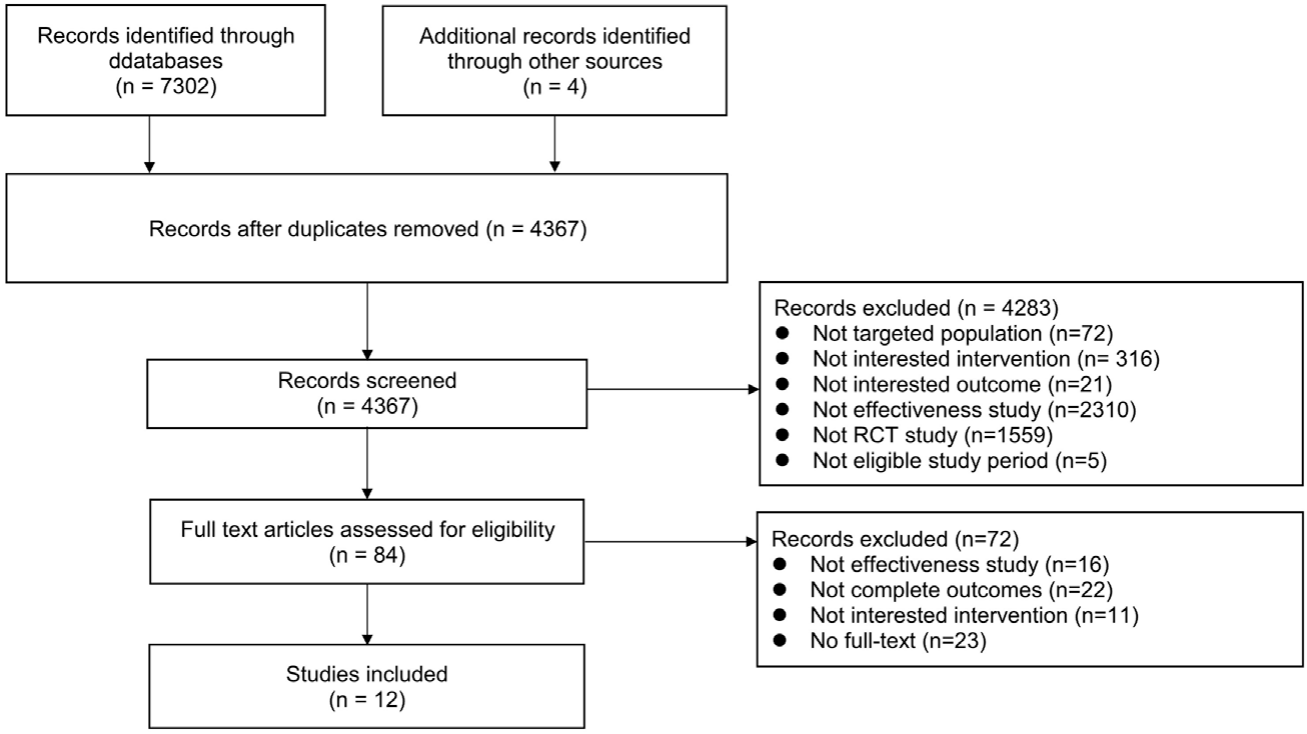
Flowchart for selection of studies.

There were 7,302 articles with 4 Supplementary Material in total according to the searching strategy. After exclusion, 12 studies involving 1,194 patients with LSS were included. Among the included studies, eight studies were from China (750 patients), two studies were from Japan (172 patients), one study was from Iran (90 patients), and one study was from Korea (182 patients). The study selection flowchart is shown in Figure 1. The basic information of all included studies is shown in Table 1, and the results of the bias risk assessment are shown in Table 2.

**TABLE 1:** Characteristics of included studies.

Study	Number of Cases	Intervention Measure		Duration/Month	Outcome
		Intervention Group	Control Group		
Haddadi et al., 2016	30/30/30	Calcitonin	Antiepileptic drugs; physiotherapy	3	②
Kim et al., 2016	60/61/61	Limaprost	Analgesics; limaprost + Analgesics	2	①②④
Wei, 2013a	35/34	Neurotrophic drugs	CTM	1	③
Wei, 2013b	60/60	Analgesics	Physiotherapy	1.25	①②
Zhenqiu, 2019	40/40	CTM	Physiotherapy	2	①③
Jian, 2013	66/69	Analgesics	Physiotherapy	1	③
Yang, 2019	30/30	Analgesics	Physiotherapy	1	①
Haishan, 2020	58/58	CTM	Physiotherapy	3	①②
Xiaoping, 2020	34/34	CTM	Physiotherapy	1	①③
Xiaoxia, 2020	51/51	CTM	Physiotherapy	1	①②
Matsuyama, 2005	67/26	Limaprost	Physiotherapy	2	①③
Takahashi et al., 2013	56/23	Limaprost	Analgesics	2	④

Note: ①,VAS; ②, ODI; ③, JOA; ④, EQ-5D.

**TABLE 2:** Bias assessment of included studies.

Studies	Method for randomization	Allocation concealment	Blinding		Completeness of outcome data	Selective outcome reporting	Other Bias
			Patients and researchers	Personnel for outcome measurement			
Haddadi et al., 2016	Low risk	Low risk	Unclear	Unclear	Low risk	Low risk	Low risk
Kim et al., 2016	Low risk	Low risk	Low risk	Low risk	Low risk	Low risk	Low risk
Wei, 2013a	Unclear	Unclear	Unclear	Unclear	Low risk	Low risk	Low risk
Wei, 2013b	Unclear	Unclear	Unclear	Unclear	Low risk	Low risk	Low risk
Zhenqiu, 2019	Low risk	Unclear	Unclear	Unclear	Low risk	Low risk	Low risk
Jian, 2013	Low risk	Unclear	Unclear	Unclear	Low risk	Low risk	Low risk
Yang, 2019	Low risk	Unclear	Unclear	Unclear	Low risk	Low risk	Low risk
Haishan, 2020	Low risk	Unclear	Unclear	Unclear	Low risk	Low risk	Low risk
Xiaoping, 2020	Unclear	Unclear	Unclear	Unclear	Low risk	Low risk	Low risk
Xiaoxia, 2020	Unclear	Unclear	Unclear	Unclear	Low risk	Low risk	Low risk
Matsuyama, 2005	Low risk	Unclear	Unclear	Unclear	Low risk	Low risk	Low risk
Takahashi et al., 2013	Unclear	Unclear	Unclear	Unclear	Low risk	Low risk	Low risk

Among all outcomes, only the VAS, ODI, JOA, and EuroQol Five Dimensions Questionnaire (EQ-5D) were available for analysis, while other outcomes could not be analyzed due to various reasons. Among the included articles, there was no article reporting any results of the VRS, SF-8, or 6-min walking distance. The NRS, the Roland Morris Disability Questionnaire (RMDQ), the MOS item short from the health survey (SF-36), and adverse effects could not be analyzed due to the limited data and unformed network. Moreover, there were various definitions of walking distance, improvement rate, and effectiveness rate in studies and therefore these outcomes were not analyzed.

### Visual Analog Scale

**FIGURE 2:**
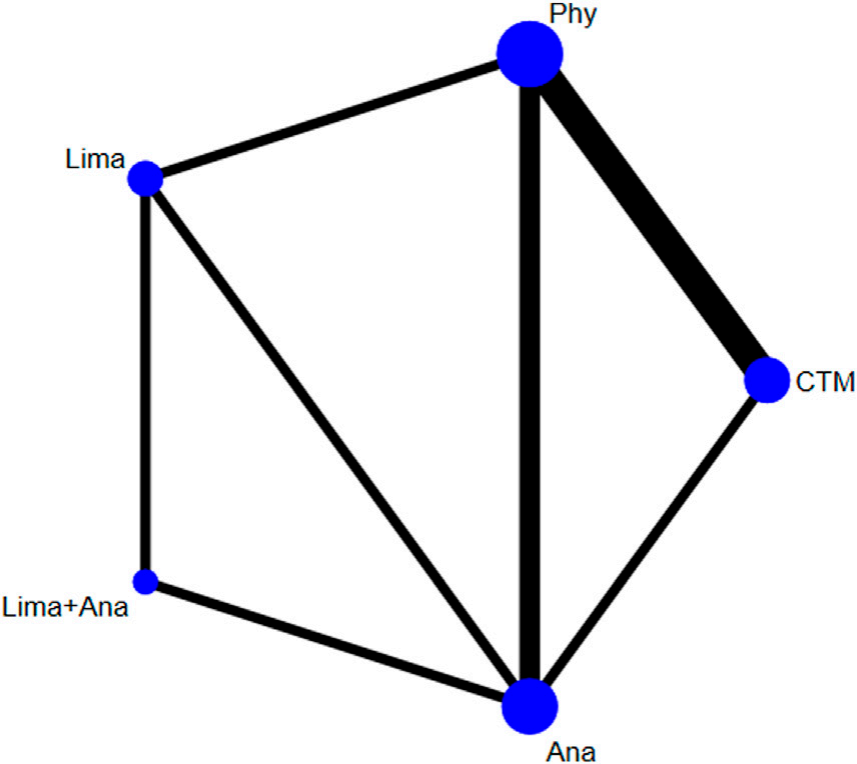
Network plot for included studies on VAS.

There were 8 studies with 821 patients reporting the VAS. The evaluated treatments included the treatment with limaprost, analgesics drugs, physiotherapy, TCM, and limaprost combined with analgesics drugs (see Figure 2). As illustrated in the figure, a larger number of studies were noted in the intervention of TCM and physiotherapy, and more patients were reported in the intervention of analgesics drugs and physiotherapy.

**FIGURE 3:**
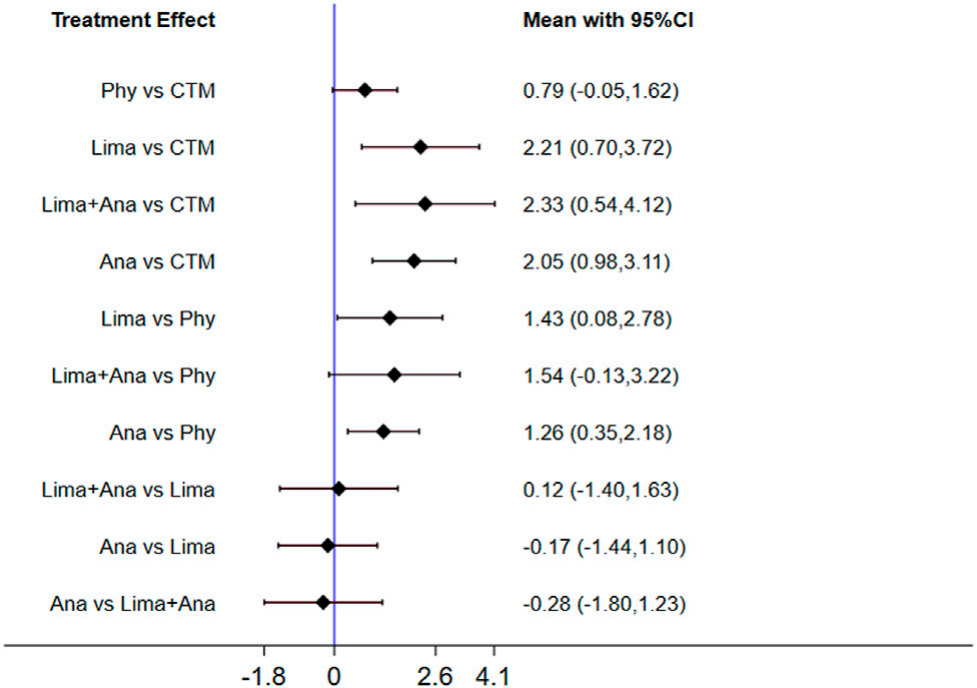
Mean difference for VAS between treatments.

The consistency model used for the analysis of the inconsistency test results was 0.07 > 0.05. The differences in interventions for reducing the VAS are shown in Figure 3. The area under the curve and the mean rank are shown in Table 3. Therefore, in terms of reducing the VAS, the ranking of efficacy was CTM > physiotherapy > analgesics drugs > limaprost > limaprost combined with analgesics drugs.

**TABLE 3:** Area under the curve and the mean rank for VAS.

Treatments	Area under the Curve (%)	Mean Rank
CTM	99.0	1.0
Phy	74.4	2.0
Ana	31.0	3.8
Lima	24.5	4.0
Lima + Ana	21.1	4.2

### Oswestry Disability Index

**FIGURE 4:**
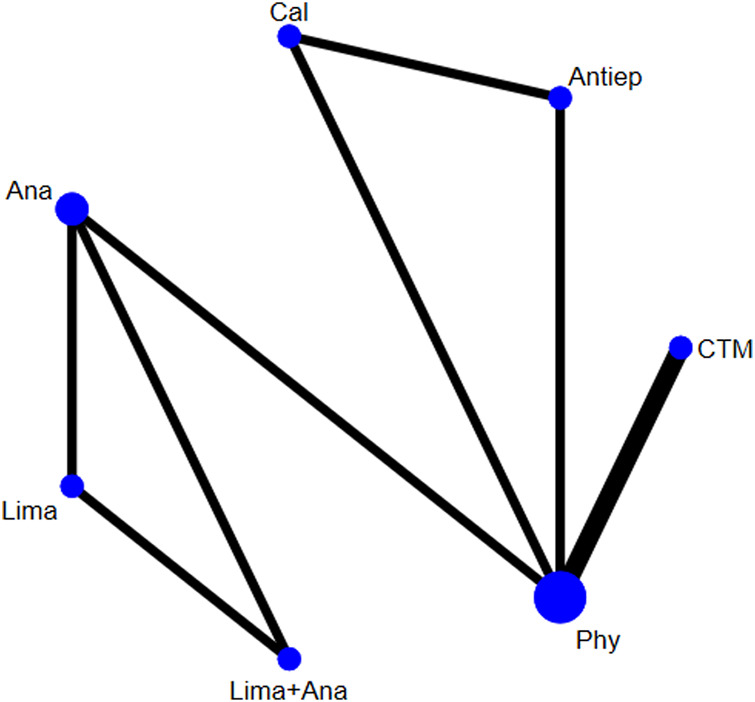
Network plot for included studies on ODI.

There were five studies with 610 patients reporting the ODI. The evaluated treatments included the treatment with calcitonin, analgesics drugs, antiepileptic drugs, limaprost, limaprost combined with analgesics drugs, physiotherapy, and CTM (see Figure 4). As illustrated in the figure, more patients were noted in the intervention of physiotherapy.

**FIGURE 5:**
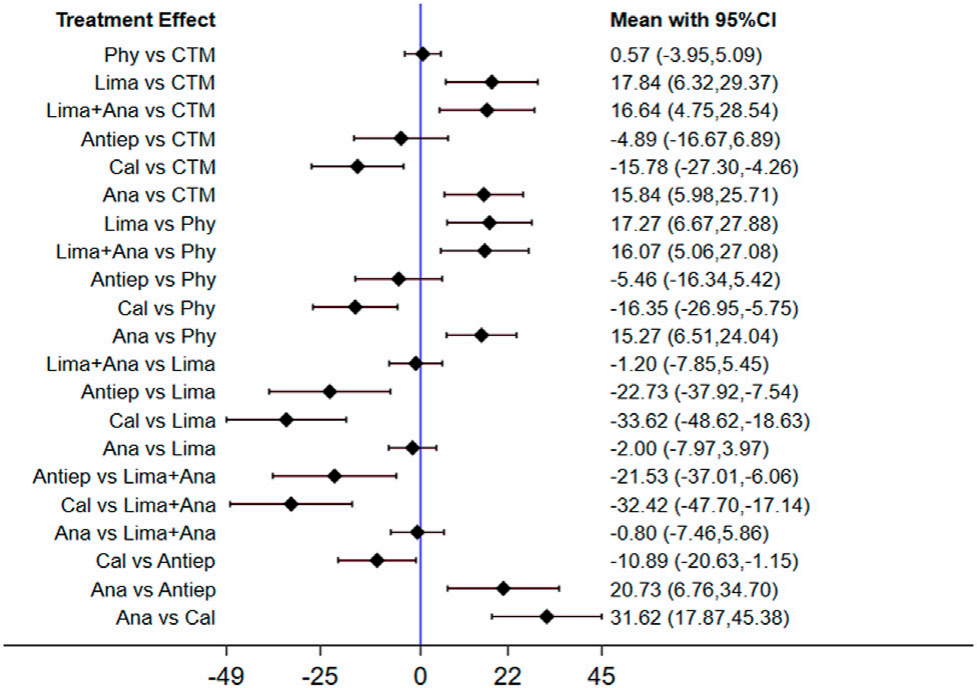
Mean difference for ODI between treatments.

The consistency model used for the analysis of the inconsistency test results was 0.81 > 0.05. The differences in interventions for reducing the ODI are shown in Figure 5, and the area under the curve and the mean rank are shown in Table 4. The results indicate that in terms of reducing the ODI, the therapeutic efficacy was ranked as limaprost > calcitonin > CTM > limaprost combined with analgesics drugs > analgesics drugs > antiepileptic drugs > physiotherapy.

**TABLE 4:** Area under the curve and the mean rank for ODI.

Treatments	Area Under the Curve (%)	Mean Rank
Lima	75.7	2.5
Cal	66.3	3.0
CTM	63.2	3.2
Lima + Ana	61.1	3.3
Ana	46.5	4.2
Antiep	44.3	4.3
Phy	26.1	5.4

### Japanese Orthopaedic Association Scores

**FIGURE 6:**
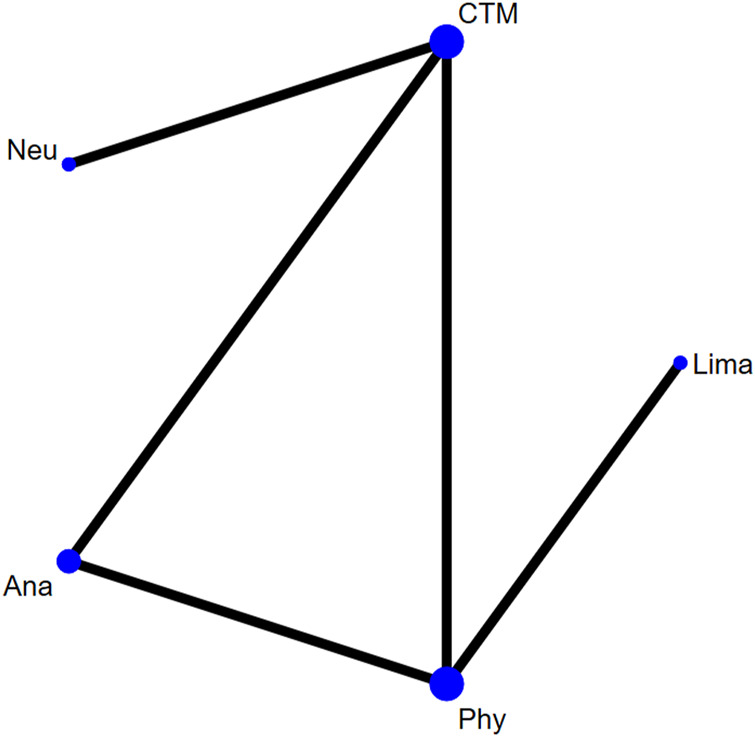
Network plot for included studies on JOA.

There were five studies with 445 patients reporting the JOA. The evaluated treatments included the treatment with analgesics drugs, limaprost, physiotherapy, neurotrophic drugs, and CTM (see Figure 6). As shown in the figure, more patients were noted in interventions of CTM and physiotherapy.

**FIGURE 7:**
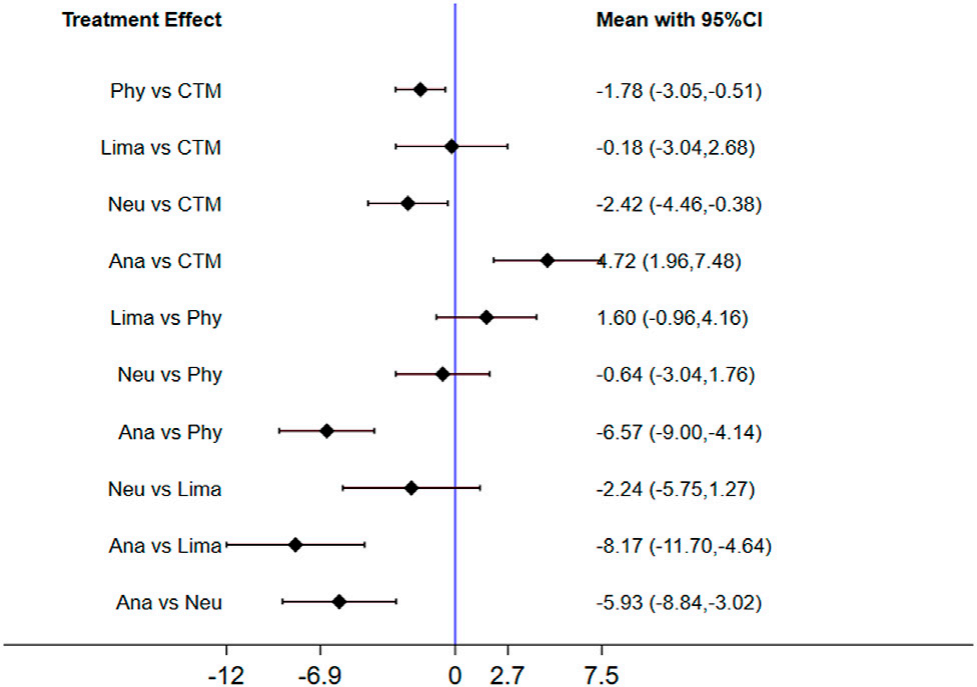
Mean difference for JOA between treatments.

The inconsistency model used for the analysis of the inconsistency test results was 0.0008 < 0.05. The differences in interventions on increasing the JOA are shown in Figure 7, and the area under the curve and the mean rank are shown in Table 5. Based on these results, the efficacy ranking was CTM > limaprost > physiotherapy > neurotrophic drugs > analgesics drugs.

**TABLE 5:** Area under the curve and the mean rank for JOA.

Treatments	Area under the curve (%)	Mean Rank
CTM	88.3	1.5
Lima	81.0	1.8
Phy	45.8	3.2
Neu	34.9	3.6
Ana	0.0	5.0

### EuroQol Five Dimensions Questionnaire

**FIGURE 8:**
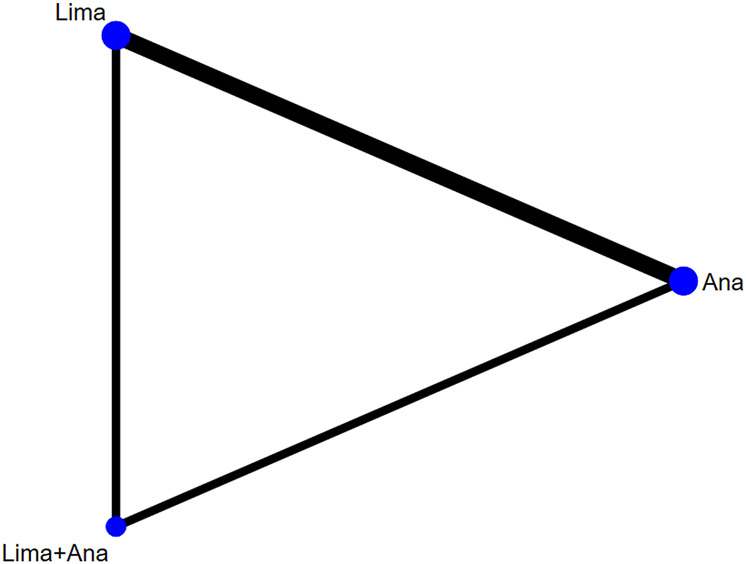
Network plot for included studies on EQ-5D.

There were 2 studies with 261 patients reporting the EQ-5D. The evaluated treatments included the treatment with limaprost, analgesics drugs, and limaprost combined with analgesics drugs (see Figure 8).

**FIGURE 9:**
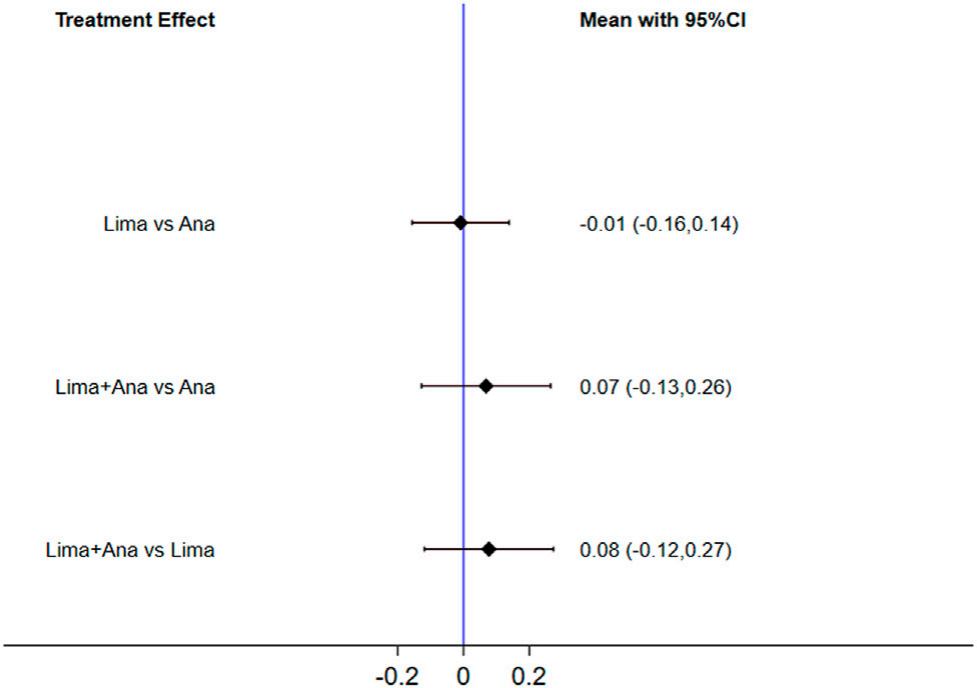
Mean difference for EQ-5D between treatments.

The consistency model fitted for the analysis of the inconsistency test results was 0.90 > 0.05. The differences in interventions on increasing the EQ-5D are shown in Figure 9, and the area under the curve and the mean rank are shown in Table 6. These results demonstrate that the efficacy ranking was limaprost combined with analgesics drugs > analgesics drugs > limaprost.

**TABLE 6:** Area under the curve and the mean rank for EQ-5D.

Treatments	Area under the curve (%)	Mean Rank
Lima + Ana	77.4	1.5
Ana	39.4	2.2
Lima	33.2	2.3

### Sensitivity Analysis

**FIGURE 10:**
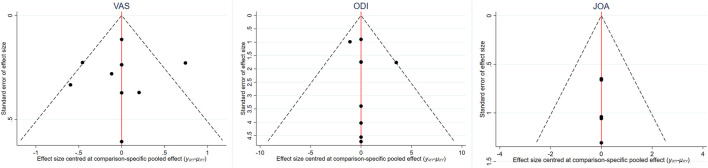
Inverted funnel chart on VAS, ODI, and JOA.

The inverted funnel charts of the VAS, ODI, and JOA were drawn to assess the publication bias, as shown in Figure 10. There was no further analysis on the EQ-5D due to the comparatively limited number of included studies using the EQ-5D as an outcome. These figures were basically symmetrically distributed, indicating the minimized publication bias among included studies. As for the clinical similarity and methodological similarity, an assessment was conducted by two reviews and the result showed that the similarity was robust and valid.

## Discussion

In the real world, the conservative treatment for patients with LSS includes pharmacological treatments like various medications and nonpharmacological treatments like exercising, massage, and so on. This study aimed to evaluate the effectiveness of the commonly used conservative treatment for patients with LSS, hoping to provide evidence for the clinical practice.

For reducing the VAS, CTM and physiotherapy were found to be the most effective treatments, which was consistent with the Chinese guideline (Group, 2014) and other studies. As mentioned in the Chinese expert consensus in 2014 (Group, 2014), physiotherapy including massage, acupuncture, electrotherapy, hyperthermia, and other treatments has relatively positive short-term efficacy than others. However, the long-term efficacy is not clear, which needs further study. A systematic review by Kim et al. (2013) evaluated the effects and safety of acupuncture, and the results showed that the acupuncture group had significantly favorable improvement over the control group on pain intensity, overall symptoms, and function outcomes related to LSS and the QoL. In this study, half of the physiotherapy was acupuncture, and all the CTM treatments were treatments combining physiotherapy and related drugs, which makes the ranking results more reasonable and comprehensible.

In terms of reducing the ODI and improving the JOA, the use of limaprost also demonstrated good efficacy than other commonly used drugs, with second ranking in the ODI and first ranking in the JOA, respectively. Up to now, limaprost is the only chemistry drug that is reported to have the specific indication of LSS, making it an important and preferred treatment in clinical practice (Matsudaira et al., 2009; Wang, 2019). As for other drugs, they are only able to relieve the symptoms, to some extent. And this superior efficacy may be due to the unique mechanism of limaprost, to reduce pain by improving the blood flow and antiplatelet aggregation in the cauda equina or nerve roots (Matsudaira et al., 2009; Wang, 2019).In addition, evidence has been accumulated for limaprost, which has been preliminarily used in Japan since the 1980s. RCT conducted by Matsudaira et al. (2009) showed that limaprost resulted in considerably greater improvements in the SF-36 subscales, and was also significantly better than etodolac for leg numbness, neurogenic intermittent claudication distance, and subjective improvement and satisfaction. Kim et al. (2016) compared the effects of limaprost and pregabalin individually and in combination for the treatment of LSS. The results showed that the efficacy of limaprost for patients with LSS was not inferior compared to that of pregabalin or the combination of limaprost and pregabalin in terms of disability.

In addition, limaprost also showed better efficacy in improving patients’ QoL. Kim et al. (2016) compared the EQ-5D scores of limaprost, pregabalin, and limaprost combined with pregabalin. The results showed that the baseline-adjusted EQ-5D scores improved significantly over time in all three groups after treatment. Takahashi et al. (2013) performed a survey to assess the relationships between the QoL and the therapy with limaprost or etodolac (an NSAID). The mean EQ-5D utility value for patients was 0.59 ± 0.12, which is much lower than the reported utility for patients with type 2 diabetes and stroke (0.86 and 0.84, respectively), suggesting that patients with LSS had poorer QoL. The EQ-5D utility value was increased by ≥ 0.1 points in significantly more patients in the limaprost group, whereas no significant changes occurred in the etodolac group.

This is the first study to evaluate the therapeutic efficacy of commonly used conservative treatments for LSS by network meta-analysis. However, there are some limitations to our study. First, the number of studies included in specific endpoints and the sample size of some studies are small, which may lead to the uncertainty of the results. Therefore, it is necessary to use further high-quality RCT studies to verify the findings of our study. Second, the clinical outcomes in this study are limited too. These results also indicate the lack of the current study in the LSS field, making it difficult for clinical practice. Further studies with long-term observation and a large sample shall be called on for better guidance and evidence support in clinics.

## Conclusion

This network meta-analysis systematically assessed the clinical effectiveness of conservative treatments in patients with LSS worldwide. Our findings demonstrated that limaprost has better efficacy in reducing the ODI, improving the JOA, and improving the QoL, while physiotherapy and CTM are found most effective in reducing the VAS in the short term after treatment. Our study provides a ranking of the therapeutic efficacy of treatments, and thus, provides the appropriate evidence to support clinical practice. However, our study still has some limitations, including the lack of studies from western countries and the small population size in included studies. Thus, further studies with a large sample and high quality are preferred to generate more valid evidence and to make up for the limitations of this study.

## Data Availability

The original contributions presented in the study are included in the article/Supplementary Material, further inquiries can be directed to the corresponding author.
