# Supplementary material for: Clinical Effectiveness of Conservative Treatments on Lumbar Spinal Stenosis: A Network Meta-Analysis
Source: Front Pharmacol. 2022 Jun 6;13:859296. doi: 10.3389/fphar.2022.859296 (PMC9207476; doi:10.3389/fphar.2022.859296)
Supplement: Supplementary file 1 [file DataSheet1.docx]

**Searching strategy of PubMed**

**#1** "lumbar spinal stenosis"[Title/Abstract] OR "LSS"[Title/Abstract] OR "DLSS"[Title/Abstract] OR "Degenerative lumbar spinal stenosis"[Title/Abstract]

**#2** "Japanese Orthopaedic Association Scores"[Title/Abstract] OR "JOA"[Title/Abstract] OR "Oswestry Disability Index"[Title/Abstract] OR "Oswestry Low Back Pain Disability Questionnaire"[Title/Abstract] OR "ODI"[Title/Abstract] OR "visual analogue scale"[Title/Abstract] OR "VAS"[Title/Abstract] OR "Verbal Rating Scale"[Title/Abstract] OR "VRS"[Title/Abstract] OR "Roland Morris disability questionnaire"[Title/Abstract] OR "RDQ"[Title/Abstract] OR "RMDQ"[Title/Abstract] OR "SF-36"[Title/Abstract] OR "EQ-5D"[Title/Abstract] OR "Patient satisfaction degree"[Title/Abstract] OR "walking distance"[Title/Abstract] OR "6 minute walking test"[Title/Abstract] OR "pain-free walking distance"[Title/Abstract] OR "maximum walking distance"[Title/Abstract] OR "improvement rate"[Title/Abstract] OR "efficiency"[Title/Abstract] OR "SF-8"[Title/Abstract]

**#3** ("2000"[Date - Publication] : "3000"[Date - Publication])

**#4** #1 AND #2 AND #3
